# The Risk Stratification for Cervical Cancer and Precursors of Domestic HPV Testing With HPV 16/18 Genotyping in Women With NILM Cytology in CentralChina: A Cohort Study

**DOI:** 10.3389/fonc.2021.716762

**Published:** 2021-10-04

**Authors:** Hui-Fang Xu, Yin Liu, Yan-Lin Luo, Dong-Mei Zhao, Man-Man Jia, Pei-Pei Chen, Meng-Jie Li, Xing-Ai Sun, Shu-Zheng Liu, Xi-Bin Sun, Shao-Kai Zhang

**Affiliations:** ^1^ Department of Cancer Epidemiology, Henan International Joint Laboratory of Cancer Prevention, Affiliated Cancer Hospital of Zhengzhou University, Henan Cancer Hospital, Henan Engineering Research Center of Cancer Prevention and Control, Zhengzhou, China; ^2^ Department of Gynecology and Oncology, Henan International Joint Laboratory of Cancer Prevention, Affiliated Cancer Hospital of Zhengzhou University, Henan Cancer Hospital, Henan Engineering Research Center of Cancer Prevention and Control, Zhengzhou, China; ^3^ Department of Pathology, Henan International Joint Laboratory of Cancer Prevention, Affiliated Cancer Hospital of Zhengzhou University, Henan Cancer Hospital, Henan Engineering Research Center of Cancer Prevention and Control, Zhengzhou, China

**Keywords:** cervical cancer, screening, HPV genotyping, risk stratification, performance

## Abstract

**Objective:**

To evaluate the clinical performance and utility for risk stratification of DH3 HPV assay in women (≥30 years) with NILM cytology.

**Methods:**

A prospective cohort was established in Central China between November 8 to December 14, 2016 which consisted of 2180 women aging 30-64 years with NILM cytology. At baseline, all women were screened using DH3 HPV assay. HPV 16/18 positive women would be assigned to colposcopy and biopsied if necessary. Then, hr-HPV positive women without CIN2+ lesions would be followed up by cytology every 12 months for two years. In the 3^rd^ year of follow up, all women that were not biopsy proven CIN2+ would be called back and screened by cytology again. In follow-up period, women with ASC-US and above were referred to colposcopy and biopsied if clinically indicated. CIN2+ was the primary endpoint in analysis. The clinical performance and utility for risk stratification of DH3 HPV assay were assessed by SPSS 22.0 and SAS 9.4.

**Results:**

Of 2180 qualified women, the prevalence of hr-HPV was 8.5% (185/2180), 45(2.1%) were HPV 16/18 positive. The clinical performance for HPV16/18 was 91.7% for sensitivity, 98.4% for specificity, respectively against CIN2+ detection at baseline. In four years of study, the corresponding rates of HPV 16/18 were 51.5% and 98.7%, respectively. The cumulative absolute risk for the development of CIN2+ was as high as 37.8% for HPV 16/18 positive women, followed by hr-HPV positive (14.6%), other hr-HPV positive (11.0%) and HPV negative (0.3%) in three years. The relative risk was 125.6 and 3.4 for HPV 16/18 positive group when compared with HPV negative and other hr-HPV positive group, respectively.

**Conclusions:**

DH3 HPV assay demonstrated excellent clinical performance against CIN2+ detection in cervical cancer screening and utility of risk stratification by genotyping to promote scientific management of women with NILM cytology.

## Introduction

Persistent high-risk human papillomavirus (HPV) infection is the necessary cause for the development of cervical intraepithelial neoplasia (CIN) and cervical cancer (1, 2). Screening has contributed much to the significant downtrend of cervical cancer burden in last decades, especially in developed countries and regions (3). HPV based screening facilitates the performance further improvement of cervical cancer screening (4, 5). Compared with cytology-based screening, HPV testing demonstrated better sensitivity against the detection of cervical cancer and precursors. Moreover, the negative predictive value (NPV) of HPV testing are also been improved for CIN3+. Hence, women with HPV negative would take a very low risk for the CINs development, which provide crucial evidences to extend the screening interval duration from 3 to 5 years in general population if HPV testing was used in the primary screening round (6). Finally, HPV testing also showed improved detection for the adenocarcinoma related lesions, which are difficult to detect for cytology-based screening (7). Due to the above excellent performance of HPV testing in cervical cancer screening, in 2018, HPV testing is recommended as an adjunct test to cytology in women with the age of 30 years and above (8).

In general, the strategies used in screening scenario are mainly based on the principle of equal management for equal risk. Although there are no universal thresholds for current strategies, the 3-year and 5-year risk for CIN3+ development which was 4.3% and 5.2%, respectively in related to low-grade squamous intraepithelial lesion (LSIL) cytology, have been identified as the benchmark for referral (9). Other study also showed that the 5-year risk is about 5-6% for CIN3+ being increasing accepted as the threshold for referral (7). Although HPV testing demonstrated better sensitivity for the detection of cervical cancer and precursor, there are still relative proportion of women were HPV positive. And it is also critical to identify the women who really take a high risk. To avoid above limitations, HPV genotyping has been proposed to increase the ability to assess the risk of women for the development of cervical cancer and precursor in compared to HPV testing with grouped HPV results. Schiffman et al. found that the cumulative 3-year risk for CIN3+ of women who were HPV 16 (10.3%) or HPV 18 (5.0%) positive, was significantly higher than those positive for any other hr-HPV type (2.3%) in women (≥30 years of age) with negative for intraepithelial lesions or malignancies (NILM) cytology (10). Therefore, stratification in this way can effectively reduce the number of women referred, with avoiding large unnecessary colposcopies.

In the light of elimination cervical cancer globally (11, 12), HPV testing with partial genotyping that could be applied widely and easily is crucial. Although the volume of commercial available HPV tests is vast, more than 90% lacks the performance evaluation steps in line with standards agreed in the HPV community in 2020 (13). Up to date, Cobas4800 HPV test and HC2 are the two mainly HPV testing that has been verified in many countries that are always been regarded as the benchmark for other HPV testing. Nevertheless, they still can’t meet the reality needs in the world, especially in developing countries. HC2 only report pooled HPV results without genotyping, and Cobas4800 needs nucleic amplification which also give rise to contaminated easily and the detection must be performed in a specific laboratory. Thus, the application of Cobas4800 was limited in resource limited areas.

DH3 HPV assay is a newly developed RNA-DNA hybrid capture-based technique that detects the presence of DNA genome of 14 hr-HPV types with HPV 16/18 genotyping at the same time, which indicated that DH3 HPV assay can be performed in a general laboratory without nucleic acid amplification. Notably, DH3 HPV assay has been reported similarly performance in compared with Cobas4800 HPV in identifying cervical cancer and precursor in cervical cancer screening (14). Nevertheless, the clinical evidences for its performance is still lacked, especially in relation to risk prediction in the follow-up. Thus, this study was designed to evaluate the clinical performance of DH3 HPV in women(≥30 years) with NILM cytology.

## Methods

### Study Design and Population

It was a prospective study conducted in Central China from November 8 to December 14, 2016. Women age 30-64 years old were enrolled according to following inclusion criteria: had intact cervix; not be screened in last three years; not be currently pregnant or within eight weeks after childbirth; had no history of hysterectomy, cervix surgery, or cervical cancer treatment; and were able to provide written informed consent. Current study was approved by the Institutional Review Board of Affiliated Cancer hospital of Zhengzhou University.

**Figure 1:**
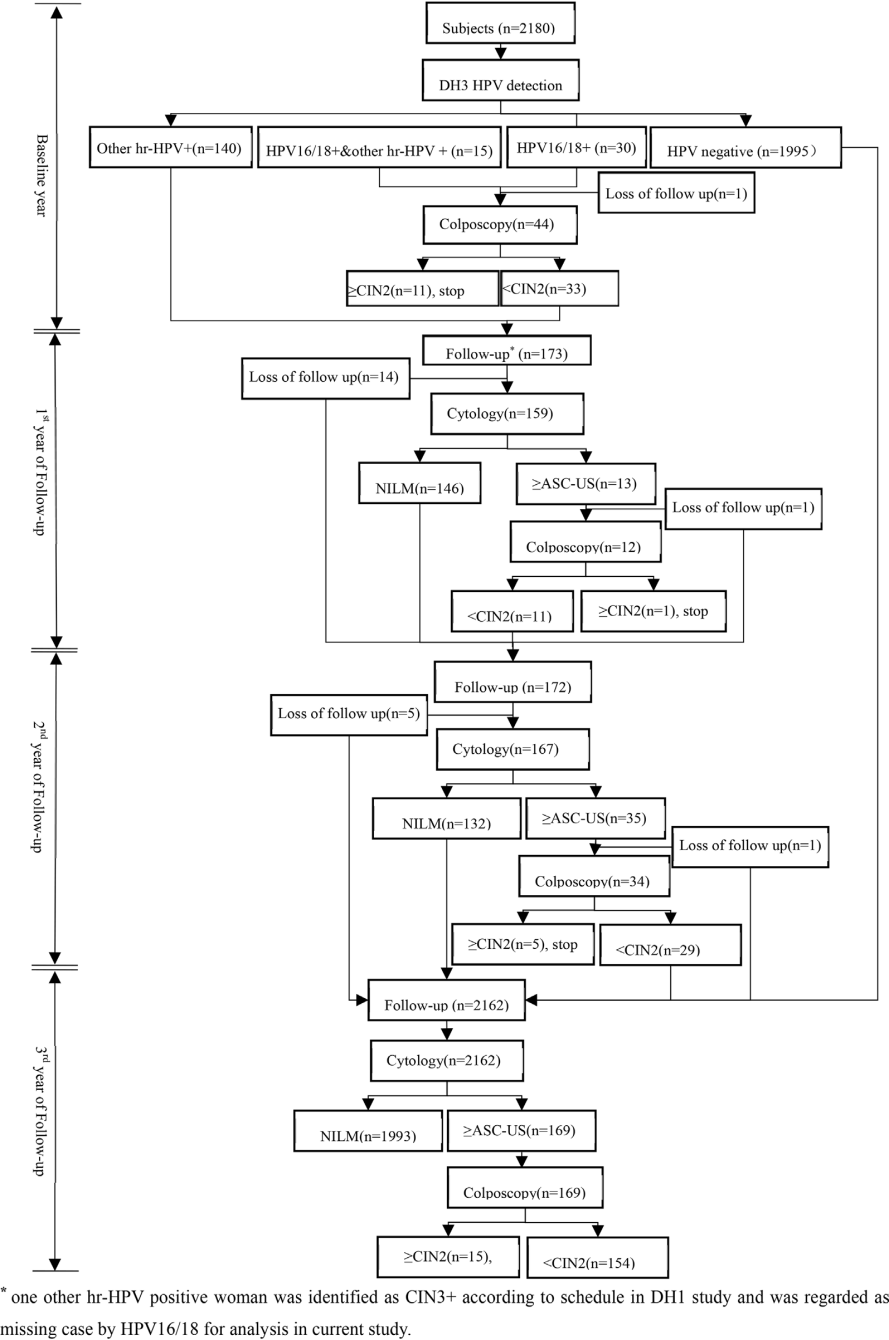
Study flowchart.

At baseline, all women would provide HPV samples that were kept in Specimen Preservation Solution (Hangzhou Dalton Biosciences, Ltd., China) for DH3 HPV assay detection after written informed consent obtained. HPV 16/18 positive women would be referred to colposcopy and biopsied if necessary. After then, all hr-HPV positive women would be rescreened by cytology every 12 months if CIN2+ was not reached in next two years. And women with ASC-US and above would undergo colposcopy and biopsied if clinically indicated. In the 3^rd^ year of follow up, all women without CIN2+ lesions would be followed up again by cytology. All biopsy specimen was fixed in 10% formalin and transferred to central laboratory for paraffin-embedded sections and diagnosis by professionals. The profile of cohort at baseline and follow up were showed in **Figure 1**.

Actually, current study overlapped part of population with another research assessed the clinical performance of DH1 in cervical cancer screening. Therefore, it is possible that CIN2+ cases could be identified by DH1 HPV assay rather than DH3 HPV assay. For such cases, they would be regarded as missing case in analysis.

### Sample Detection

In current study, cytological and HPV samples were collected for screening during baseline and follow up period.

#### Cytology

In current study, all cytological samples were evaluated according to the TBS (The Bethesda System) system. Each satisfactory cytological sample would be diagnosed by cytologist as following results: negative for intraepithelial lesion or malignancy (NILM), atypical squamous cells undetermined significance (ASC-US), atypical squamous cells‐cannot exclude HSIL (ASC-H), low squamous intraepithelial lesion (LSIL), high squamous intraepithelial lesion (HSIL), squamous of cervical carcinoma (SCC), atypical glandular cells(AGC). Women with ASC-US and above were determined as abnormal cytological results and would be further examinations.

#### HPV Detection

All HPV samples would be detected by DH3 HPV assay, a novel domestic high-risk HPV testing based on hybrid capture. Firstly, the denatured samples were mixed with probe A (probe cocktail containing RNA probes for HPV16 and 18) and probe B (probe cocktail containing RNA probes for HPV31, 33, 35, 39, 45, 51, 52, 56, 58, 59, 66, and 68) in two microplates. The probes targeted the entire HPV genome. The resultant RNA-DNA hybrids were captured by specific antibodies, immobilized on a microplate, and further detected by alkaline phosphatase-conjugated antibodies specific for the RNA-DNA hybrids. As the substrate is cleaved by alkaline phosphatase, light is emitted and measured as relative light unit (RLU) using an illuminometer. The intensity of the emitted light indicates the viral load of the target HPV in the sample. The cut-off value was 1.0 pg/ml for DH3 HPV assay as the threshold indicating HPV-positivity in according to the guidelines of World Health Organization (WHO). In current study, all HPV detection was strictly conducted in accordance with the standard operation protocol.

### Statistical Analysis

In current research, categorical variables were showed as proportions and compared using χ^2^ tests. Continuous variables were described as mean (SD) or median (IQR). The clinical performance of DH3 HPV assay would be assessed using sensitivity, specificity, positive predictive value (PPV), negative predictive value (NPV), respectively, based on the HPV result at baseline. The cumulative absolute risk and relative risk for the development of cervical cancer and precursor in three years were also evaluated for different HPV results. And the corresponding 95% confidence interval (95%CI) would also be calculated for clinical performance and risk evaluation. In current study, CIN2+ was used as the clinical endpoint. Statistical significance of all two-tailed tests was set at *P* ≤ 0.05. The SPSS 22.0 and SAS 9.4. were used for the statistical analysis.

## Results

In current study, 2444 enrolled women were invited but 37 were excluded because of hysterectomy, cervix surgery, or cervical cancer treatment, and 227 were dropped in follow up period. Therefore, 2180 were qualified for analysis, and the mean age was 47.3 ± 7.5 years.

At baseline, 45 were HPV 16/18 positive (2.1%), at the same time, 155 were positive (7.1%) for any other hr-HPV type. At baseline, 5 CIN2 cases and 7 CIN3+ cases were identified. In follow-up period, 12 CIN2 cases and 9 CIN3+ cases were found, respectively, of which 6 CIN2+ cases in the 3^rd^ year of follow up were HPV negative at baseline (**Table 1**).

**Table 1:** Distribution of CIN2+ and CIN3+ cases at baseline and follow up period.

DH3	Cases (N)	Baseline (n)		1^st^ year (n)			2^nd^ year (n)			3^rd^ year (n)	
		CIN2	CIN3+	CIN2	CIN3+		CIN2	CIN3+		CIN2	CIN3+
HPV 16/18+	30	2	6	0	0		1	0		1	0
HPV 16/18& Other HPV+	15	3	0	0	0		1	0		1	2
Other HPV+	140	0	1	1	0		1	2		3	2
HPV negative	1995	0	0	0	0		0	0		3	3
**Total**	2180	5	7	1	0		3	2		8	7

CIN, Cervical intraepithelial neoplasia.

At baseline, the sensitivity and specificity of DH3 were 100% and 92.0% against CIN2+ detection for hr-HPV positive. In contrast, DH3 showed a sensitivity of 91.7% and a specificity of 98.4% for HPV 16/18 positive. If using CIN3+ as clinical endpoint, they were 100%, 91.8%, and 85.7%, 98.2%, respectively. Considering all cases identified, the overall sensitivity and specificity achieved 81.8% and 92.6% for hr-HPV positive against CIN2+ detection. Similarly, they were 51.5% and 98.7% for HPV 16/18 positive. For CIN3+ detection, the corresponding rates were 81.3%, 92.0%, 50.0% and 98.3%, respectively (**Table 2**).

**Table 2:** Performance of DH3 HPV assay in cervical cancer screening.

DH3		Cases	Non-cases	Sensitivity	Specificity	PPV	NPV
**Baseline**						
**CIN2+**						
**Any HPV**						
HPV+	12	173	100 (75.8, 100)	92.0 (90.8, 93.1)	6.5 (3.8, 1.0)	100.0 (99.8, 100.0)
HPV-	0	1995				
**HPV 16/18**						
HPV 16/18+	11	34	91. 7 (64.6, 98.5)	98.4 (97.8, 98.9)	24.4 (14.2, 38.7)	100.0 (99.7, 100.0)
HPV 16/18-	1	2134				
**CIN3+**						
**Any HPV**						
HPV+	7	178	100.0 (64.6, 100.0)	91.81 (90.6, 92.9)	3.8 (1.9, 7.7)	100.0 (99.9, 100.0)
HPV-	0	1995				
**HPV 16/18**						
HPV 16/18+	6	39	85.7 (48.7, 97.4)	98.2 (97.6, 98.7)	13.3 (6.3, 26.2)	100.0 (99.7, 100.0)
HPV 16/18-	1	2134				
**Cumulative performance in three years**						
**CIN2+**						
**Any HPV**						
HPV+	27	158	81.8 (65.6, 91.4)	92.6 (91.5, 93.7)	14.6 (10.2, 20.4)	99.7 (99.4, 99.9)
HPV-	6	1989				
**HPV 16/18**						
HPV 16/18+	17	28	51.5 (35.22, 67.5)	98.7 (98.12, 99.1)	37. 8 (25.11,52.37)	99.3 (98.8, 99.5)
HPV 16/18-	16	2119				
**CIN3+**						
**Any HPV**						
HPV+	13	174	81.3 (57.0, 93.4)	92.0 (90.8, 93.1)	7.0 (4.2, 11.7)	99.9 (99. 6, 100.0)
HPV-	3	1992				
**HPV 16/18**						
HPV 16/18+	8	37	50.0 (28.0, 72.0)	98.3 (97.7, 98.8)	17.8 (9.3, 31.33)	99.6 (99.3, 99.8)
HPV 16/18-	8	2127				

CIN, Cervical Intraepithelial Neoplasia; PPV, Positive predictive value; NPV, Negative predictive value.

The performance of DH3 for risk prediction was showed in **Table 3**. For HPV results at baseline, HPV 16/18 positive group had the highest cumulative absolute risk for the development of CIN2+ lesions in three years (37.8%, 95%CI: 25.1% to 52.4%), followed by hr-HPV positive group (14.6%, 95%CI: 10.2% to 20.5%). In contrast, the risk of HPV negative group was the lowest (0.3%, 95%CI: 0.1% to 0.7%). For the relative risk against CIN2+ detection, HPV 16/18 positive group was 125.6(95%CI: 52.0, 303.6) times higher than that of HPV negative group. Similarly, they were 48.5(95%CI: 20.3, 116.0) times higher for hr-HPV positive group and 36.5 (95%CI: 14.6, 91.2) times higher for other hr-HPV positive group when compared with that of HPV negative group, respectively. If compared with other hr-HPV positive group, HPV 16/18 positive group had 3.4(95%CI: 1.9, 6.2) times higher relative risk. For CIN3+ detection, HPV 16/18 positive group had the highest cumulative absolute risk in three years (17.8%, 95%CI: 9.0% to 31.6%), followed by hr-HPV positive group (7.0%, 95%CI: 4.1% to 11.8%). And the HPV negative group had the lowest absolute risk for CIN3+ detection (0.2%, 95%CI: 0.0% to 0.5%). In compared with HPV negative group, HPV 16/18 positive group had a 118.2 (95%CI: 32.4, 431.0) times higher relative risk. Meanwhile it was 46.7(95%CI: 13.4, 162.5) times higher for HPV positive group, and 30.0 (95%CI: 7.8, 115.0) times higher for any other hr-HPV positive group. And in compared with other hr-HPV positive group, the relative risk was 3.9(95%CI: 1.5, 10.3) times higher for HPV 16/18 positive group.

**Table 3:** The performance for risk prediction in cervical cancer screening.

DH3	Cumulative absolute risk (%, 95CI%)	Relative risk (95%CI)	
		HPV-	Other HPV+
**CIN2+**			
Any HPV+	14.6 (10.2, 20.5)	48.5 (20.3, 116.0)
HPV 16/18+	37.8 (25.1, 52.4)	125.6 (52.0, 303.6)	3.4 (1.9, 6.2)
Other HPV+	11.0 (6.9, 17.0)	36.5 (14.6, 91.2)
HPV negative	0.3 (0.1, 0.7)			
**CIN3+**			
Any HPV+	7.0 (4.1, 11.8)	46.7 (13.4, 162.5)
HPV 16/18+	17.8 (9.0, 31.6)	118.2 (32.4, 431.0)	3.94 (1.5, 10.3)
Other HPV+	4.5 (2.0, 9.2)	30.0 (7.8, 115.0)
HPV negative	0.2 (0.0, 0.5)

## Discussion

Current study focused on women 30 years or older with NILM cytology. The main findings demonstrated even for NILM women, their absolute and relative risks to develop CIN2+ and CIN3+ were associated closely with hr-HPV infection status, especially whether infected HPV16/18. Therefore, HPV16/18 genotyping could stratify the risk of cervical cancer and precursor, and distinguish high risk population effectively in women with NILM cytology, indicating that the novel hybrid capture HPV test, DH3 HPV assay, had great potential strength in cervical cancer screening, especially in resources limited areas.

Here, the prevalence of overall HPV (8.5%) was similar to those findings reported or other FDA-approved HPV assays in women with NILM cytology, including the Onclarity HPV assay (9) and LA-HPV assay (15), which reported a prevalence of 7.9% and 9.0%, respectively. For individual HPV genotypes HPV 16/18, the ATHENA trial reported similar prevalence values for both HPV 16 (1.3%) and HPV 18 (0.6%) as those reported here (2.1%). The findings also revealed that the prevalence of HPV 16/18 in CIN2+ was significantly higher than any other hr-HPV types. Therefore, the results here are consistent with the findings derived in previous studies (15–19).

The clinical performance of any hr-HPV and HPV 16/18 was good at baseline because of study design. All CIN2+ cases were hr-HPV positive, but only one CIN3 case was other hr-HPV positive at baseline. Therefore, HPV 16/18 genotyping had a sensitivity of 91.7% for CIN2+ detection, 85.7% for CIN3+ detection. The corresponded specificity was 98.4% and 98.2% respectively. Meanwhile, the overall sensitivity decreased to around 80% for hr-HPV, 50% for HPV 16/18. For specificity, it is about 92% and 98%, correspondingly, if considering all cases found during baseline and follow and period. The performance of DH3 in current research for the detection of CIN2+ and CIN3+ is similarly to those findings of an established, clinically validated HPV assay (HC2) which had a sensitivity of 98.7%, and a specificity of 94.1% for CIN2+ detection (20). However, in compared with HC2, DH3 HPV assay made the partial genotyping possible which provided strong evidence that HPV detection and genotyping could make improvement on the clinical utility as an adjunct test with cytology in the practice of cervical cancer screening. Also, the performance of DH3 HPV assay was largely in agreement with Onclarity HPV assay, which were reported a sensitivity of 87.5%, and a specificity of 48.6% against the detection of CIN2+ (93.5% and 48.3% for CIN3+) (9).

For the risk estimation, Uijterwaal MH and colleagues made a five-year risk assessment for the development of CIN2+ or CIN3+ in 2015 (21). In their study, NILM women with hr-HPV positive take a risk of 7. 9% (95%CI: 4.4%, 10.1%) for CIN3+, 12.9% (95%CI: 9.6%, 16.0%) for CIN2+ in five years. For women with NILM cytology and HPV 16/18 positive, the corresponding risk were 18.1% (95%CI: 9.4%, 33.9%) and 24.6% (95%CI: 16.7%, 30.2%). Compared with current study, the five-year risk for CIN2+were lower but the higher for CIN3+. Nevertheless, the risk in current study was a three-year risk.

As other studies found, the risk of women with HPV negative and NILM cytology was very low, in current research, they were 0.3% and 0.2% in three years, indicating that the DH3 HPV assay can effectively predict elevated risk for both CIN2+ and CIN3+ in women at the age of 30 years or older with NILM cytology who were considered to take an extremely low risk before. Finally, these results show that the differential stratification of risk through the detection of individual genotypes (HPV16/18) has the potential to affect patient care pathways in this population. Furthermore, the findings described here are similarly to the established risk thresholds for non–HPV16/18 (22).

Prior cross-sectional and prospective studies have clearly demonstrated that hr-HPV status was an important predictor of the current and future risk for the occurrence of CIN2+ in women with NILM cytology (23). To make clinical decision scientific and effective during cervical cancer screening and management, equal management for equal risk has been regarded as the guiding principle (16, 24). In current study, hr-HPV carry an cumulative absolute risk of 7.0% for the detection of CIN3+, especially 17.8% of HPV 16/18 positive group would be CIN3+, which were above the 5-year risk threshold for referral to colposcopy (5–6%; based on risk values associated with LSIL cytology or ASC-US and HPV positive results) (16, 24, 25). These findings indicated that the screening interval for women with NILM cytology may be better if less than three years in China, and additional reflex is necessary even in NILM cytology women. Nevertheless, some other factors should also be considered when policy makers set the screening schedule (e.g., the risk thresholds for clinical action, the number of women assigned to colposcopy, and the health resources available). And this study provides robust evidenced that, with NILM cytology, HPV 16/18 genotyping was a promising reflex measure in the management of NILM population. However, the findings here still need to be verified in large population.

Presently in US clinical practice, acceptable actions following an hr-HPV positive result in the NILM population during screening include either repeat co-testing after 1 year or concurrent HPV genotyping. For the latter step, HPV 16/18 positive women would be directed to colposcopy, whereas HPV 16/18 negative women would undergo repeat co-testing in 1 year. However, it is often underappreciated that HPV positive women with NILM cytology fall below the risk threshold for colposcopy (CIN2+ as the clinical endpoint). Current US guidelines regarding co-testing for women ≥30 years of age suggest only HPV 16/18 positive women with NILM be referred based on the risk of cervical cancer and precursor (24, 26, 27). Because HPV 16 and 18 are the two most prevalent oncogenic genotypes in cervical cancer which could be responsible for 61-84.5% of cervical cancer cases (1, 28–30) and HPV 16/18 was associated with a high baseline risk (24.4%) for high-grade cervical disease among women with NILM cytology (17).

DH3 HPV assay is a newly developed HPV test based on the hybrid capture technology. Similar to HC2, DH3 HPV assay could detect the presence of 14 hr-HPV by magnifying and detecting the chemical signal without nucleic acid amplification, which make it possible for the assay to be performed in a general laboratory that is more practicable in resource limited areas (31). But unlike HC2, DH3 HPV assay separately detects HPV16/18 and 12 other hr-HPV types by two different patented probe cocktails. Thus, DH3 made it possible to directly referred HPV16/18 positive women to colposcopy and improve the performance of population management in cervical cancer screening.

In summary, the performance and risk detection obtained using DH3 HPV assay supports established screening algorithms within the current cervical cancer screening guidelines for co-testing and demonstrates its utility for the triage of women with NILM cytology in cervical cancer screening.

## Data Availability Statement

The raw data supporting the conclusions of this article will be made available by the authors, upon reasonable request.

## Ethics Statement

The studies involving human participants were reviewed and approved by The Institutional Review Board of Affiliated Cancer Hospital of Zhengzhou University. The patients/participants provided their written informed consent to participate in this study.

## Author Contributions

H-FX drafted the manuscript. YL completed the statistical analyses. Y-LL and D-MZ were responsible for the gynecological examination and diagnosis. P-PC, X-A, and M-JL collected the samples and information. M-MJ managed the database. X-BS and S-KZ were supervised the study. S-KZ revised the manuscript substantially. All authors contributed to the article and approved the submitted version.

## Conflict of Interest

The authors declare that the research was conducted in the absence of any commercial or financial relationships that could be construed as a potential conflict of interest.

## Publisher’s Note

All claims expressed in this article are solely those of the authors and do not necessarily represent those of their affiliated organizations, or those of the publisher, the editors and the reviewers. Any product that may be evaluated in this article, or claim that may be made by its manufacturer, is not guaranteed or endorsed by the publisher.
